# Public-private partnerships to build human capacity in low income countries: findings from the Pfizer program

**DOI:** 10.1186/1478-4491-5-8

**Published:** 2007-03-02

**Authors:** Taryn Vian, Sarah C Richards, Kelly McCoy, Patrick Connelly, Frank Feeley

**Affiliations:** 1 Center for International Health and Development, Boston University School of Public Health, 715 Albany Street, T4W, Boston, MA, USA; 2 Independent Consultant, 522 Haverhill Road, Chester NH, USA

## Abstract

**Background:**

The ability of health organizations in developing countries to expand access to quality services depends in large part on organizational and human capacity. Capacity building includes professional development of staff, as well as efforts to create working environments conducive to high levels of performance. The current study evaluated an approach to public-private partnership where corporate volunteers give technical assistance to improve organizational and staff performance. From 2003 to 2005, the Pfizer Global Health Fellows program sent 72 employees to work with organizations in 19 countries. This evaluation was designed to assess program impact.

**Methods:**

The researchers administered a survey to 60 Fellows and 48 Pfizer Supervisors. In addition, the team conducted over 100 interviews with partner organization staff and other key informants during site visits in Uganda, Kenya, Ghana, South Africa and India, the five countries where 60% of Fellows were placed.

**Results:**

Over three-quarters of Fellowships appear to have imparted skills or enhanced operations of NGOs in HIV/AIDS and other health programs. Overall, 79% of Fellows reported meeting all or most technical assistance goals. Partner organization staff reported that the Fellows provided training to clinical and research personnel; strengthened laboratory, pharmacy, financial control, and human resource management systems; and helped expand Partner organization networks. Local staff also reported the Program changed their work habits and attitudes. The evaluation identified problems in defining goals of Fellowships and matching Organizations with Fellows. Capacity building success also appears related to size and sophistication of partner organization.

**Conclusion:**

Public expectations have grown regarding the role corporations should play in improving health systems in developing countries. Corporate philanthropy programs based on "donations" of personnel can help build the organizational and human capacity of frontline agencies delivering health services. More attention is needed to measure and compare outcomes of international volunteering programs, and to identify appropriate strategies for expansion.

## Background

As international donors expand global financing in response to the HIV-AIDS pandemic and in support of the Millennium Development Goals, issues of human and organizational capacity are becoming increasingly important [1]. Governmental and non-governmental organizations alike are dealing with important capacity constraints which prevent services from being delivered in sustainable ways. Challenges include highly visible constraints on number, distribution, and training of service delivery staff, especially in countries hardest hit by the HIV-AIDS epidemic [2-4]. Less visible, but no less important, are capacity problems related to health systems operational efficiency, productivity, process improvement, and sustainability [5]. The capacity to continuously address new problems and improve access to quality health services requires not only financial and material inputs, but also investments in leadership development, management, and service delivery systems improvement [6,7].

The private business sector is recognized as an important stakeholder in international development, especially in the health sector. Public-private partnerships are being pursued as a way to leverage ideas, resources, and capabilities to achieve public health goals [8]. Most commonly, business sector contributions to capacity building in the health sector have included philanthropic donations of essential inputs such as drugs and financial resources [9-11]. Research-based pharmaceutical companies gave over $564 million in donations to developing countries in 2001 [12], and drug donation programs launched by Merck, SmithKline Beecham, Pfizer, and Beckton Dickinson have been helpful in improving access to health care for more than 248 million people [13]. Drug donation programs have strengthened organizational capacity for communicating and collaborating with development partners, and helped to develop new systems for drug distribution and monitoring & evaluation. At the same time, however, such programs may pose challenges in regard to ownership, accountability, and respect for due process [9].

In workshops organized by the Global Health Initiative of the World Economic Forum to discuss how business can help strengthen service delivery capacity in Sub-Saharan Africa, management was identified as a key challenge amenable to solutions sought in partnership with business [6]. One type of program designed to build management capacity for service delivery in developing countries is international volunteering. Traditionally organized by professional, faith-based, and non-profit organizations [14], volunteering programs are increasingly being offered by businesses, either alone or in association with other businesses [15,16]. For example, the Brookings Institute recently created an Initiative on International Volunteering and Service with representatives from major multinational corporations such as IBM, Microsoft and Pfizer [17]. In Europe, public-private partnerships such as the Netherlands PUM program and the Dutch Employers Cooperation Programme (DECP) have been fielding business volunteers to build capacity of local business organizations in developing countries [18,19], while the international transportation giant TNT has had a public-private partnership with the UN World Food Programme since 2002, offering technical specialist volunteers for up to one year to help build capacity through knowledge transfer, mentoring, and other projects [20]. Yet, little is known about the impact of these programs on organizations receiving assistance, especially in the developing world.

In 2005, Boston University's Center for International Health and Development was asked by the U.S. Agency for International Development (USAID) and Pfizer Corporation to undertake an evaluation of Pfizer's international volunteering program. From 2003 to 2005, Pfizer's Global Health Fellows (GHF) Program placed 72 employees in organizations based in 19 countries. The goal of the program was to promote better health by improving the service delivery capacity of local partners in poor countries. The Boston University research team sought to determine the impact of Pfizer's international volunteering program on the organizations receiving assistance, and to document lessons learned for public-private collaboration in capacity building.

### Program history and structure

Pfizer's Global Health Fellows Program matches qualified and interested Pfizer staff with assignments in local partner organizations in the developing world. The Program identifies potential local partners and Fellowship assignments through intermediary non-governmental organizations (NGOs) such as Health Volunteers Overseas (HVO) and American Jewish World Services (AJWS). Pfizer also works directly with large international NGOs such as Family Health International, Médecins du Monde, and other partner organizations such as the Uganda Infectious Disease Institute.

Twice a year, Pfizer employees apply for selection as Fellows through a competitive process. The approval of a fellow's supervisor must be obtained in order for them to participate in the program, which implies that the Fellow's work will be covered while overseas. The Pfizer work unit Supervisor is not responsible for the matching process or program impact, though Supervisors communicate with Fellows during the Fellowship.

Partner Organizations review Fellowship candidates for a defined assignment, and select a Fellow from the candidate pool. Fellows receive pre-departure training and information concerning health issues, security, cultural competence, and GHF Program procedures. They also are encouraged to contact the Partner Organization to discuss their scope of work prior to arrival.

Assignments average three to six months. The fellow's salary and benefits are charged to her/his Pfizer work unit, while living allowance and travel are paid by Pfizer Corporate Philanthropy, which also pays a small allowance to the Partner Organization for supervision. Subject to approval, the Fellow may also use up to $500 to cover material costs for local projects.

**Figure 1:**
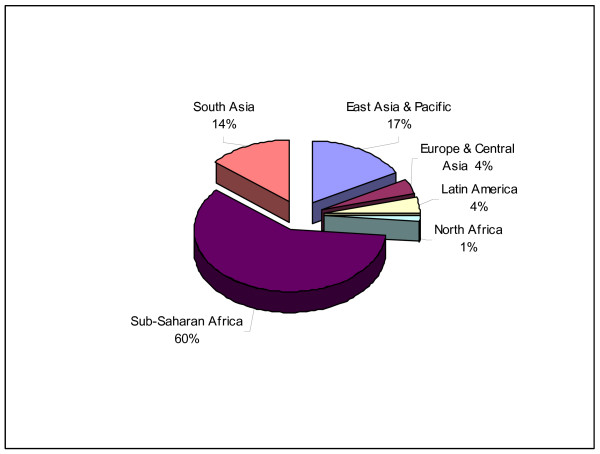
Geographic distribution of Pfizer Global Health Fellows as of October 2005.

Figure 1 shows the geographic distribution of Fellowships as of October 2005, illustrating the concentration of Fellows in Sub-Saharan Africa (60%).

### Evaluation design

Conducted between October 2005 to January 2006, the evaluation sought to answer the following questions:

▪ How do Fellowships affect the Partner Organizations with which Fellows are placed? How does the Fellowship Program build institutional and professional capacity?

The evaluation also examined how the Fellowships affect participating Pfizer employees and their work units, and examined the effect of the Global Health Fellows Program on Pfizer's reputation and corporate activities (findings presented elsewhere) [21]. This article presents the findings related solely to the impact on Partner Organizations.

## Methods

The evaluation team used both qualitative and quantitative methods to collect data. Two approaches were used: a survey for eliciting information from Fellows and Pfizer Supervisors, and structured in depth interviews to gather information from Partner Organizations. The diversity of respondents within Partner Organizations (co-workers, supervisors, program directors, and beneficiaries, among others) made it difficult to use the survey approach. We also wanted to explore perceptions of program goals and impact in more detail through the in-depth interviews with Partner respondents. The study design was approved by the Boston University Medical Campus Institutional Review Board.

### Fellow and supervisor surveys

We surveyed Fellows (both returned and current as of January 2006) and the Pfizer Supervisors of Fellows. We attempted to contact all 72 Fellows and 69 Supervisors who had participated in the program as of October 2005, including those who had subsequently left the company. Our response rates were 83% (60) for Fellows and 70% (48) for Supervisors. Fifteen of the 33 non-respondents did not answer repeated e-mails and phone messages, 14 had missing contact information, and 4 were interviewed too late to be included in the analysis. About 72% of Fellows interviewed were female, which is the same percentage as in the total population of Fellows. Interviews were conducted in person or by telephone. The survey questions were arranged by phase of fellowship (prior to, during, and post) with questions to elicit suggestions and comments, and to record interviewer observations. Confidentiality was assured by using codes to identify respondents, and by masking identifying detail from qualitative responses.

Fellows were asked questions about preparation, goals and scope of work, achievements, and impact of the program on themselves, the Partner Organization, their Pfizer workgroup, and the company as a whole. Supervisors of Fellows were asked about reasons for approving the Fellowship, preparation, how work was covered in the Fellow's absence, and the impact of the program on the Fellow, the Pfizer workgroup, and the company. Partner Organization respondents were asked about goals and scope of work, achievements, and the impact of the program on the Partner Organization, and their impressions of the company.

Qualitative data from the surveys were entered into NVivo 2.0 software. NVivo is a computer program that allows for more complex categorization of large sets of qualitative data than when done manually. Domain analysis was subsequently conducted by a research team member and assistant. Quantitative data were analyzed using Microsoft Excel and SAS v9.1 to produce frequency tables and means where appropriate. Data were stratified by round of Fellowship and current versus returned Fellows to elicit trends.

### NGO Partner interviews

Researchers also interviewed personnel from Partner Organizations in Kenya, Uganda, Ghana, South Africa, and India – countries which represented 60% of Fellowships. We interviewed the manager of the Organization where each Fellow was placed, the Fellow's immediate Supervisor, and co-workers. In a limited number of cases, we were able to interview local recipients of the services the Fellow was attempting to improve. For many Fellowships, we visited the work site and in some cases reviewed the systems changes resulting from the Fellow's recommendations.

In total, the research team conducted over 100 NGO Partner interviews. Members of the evaluation team wrote notes from each interview, and all references to individual Fellows were coded using the same coding system used to identify Fellow interviews. In order to encourage full disclosure, respondents were promised that feedback on individual Fellows would not be shared with the Fellow or with Pfizer except as part of our aggregate findings. The interview notes were shared with all five members of the study team. Where appropriate, respondent's impressions of Fellowship accomplishments were compared with statements made by the Fellows. This comparison has informed our general conclusions, but no specific contrasts are shown in our results in order to maintain confidentiality.

The evaluation team met after completion of all field interviews to formulate general conclusions from the interview material. In our discussions, we developed categories for grouping Fellowships, based on level of skill/judgment applied by the Fellow and the level of impact on the host organization and the services it provides. This process is described in more detail in the Results section.

## Results

### Fellowship goals

Knowledge transfer was an overriding theme in Fellows' statements of their professional goals, while on a personal basis many Fellows noted a desire to "do good", serve the poor, and to grow personally. Fellows described their intention to make a positive impact on the Partner through transferring their expertise. Fellows also described what they believed were their Partner Organization's goals, which the research team grouped into six broad categories, shown in Table 1. Most commonly, Partner goals focused on management systems improvements, assistance in organizational planning, training, and evaluation.

**Table 1:** NGO Partner goals as articulated by Global Health Fellows

**Category**	**Illustrative goals**	**Number (%) of Fellows who said this was an NGO goal**
Management and planning	Strengthening management of facilities, systems, or data; process or quality improvement; strategic, organizational or human resources planning; communication or marketing plan development	20 (34%)
Training and education	Mentoring; training NGO staff; teaching students; educating community members	19 (32%)
Documentation	Writing or revising Standard Operating Procedures (SOP) or best practices; grant writing; producing publications	17 (29%)
Evaluation	Evaluate an existing or proposed program; conduct assessment of staff	12 (20%)
Technical or scientific capacity building	Software installation, database creation, laboratory or clinic set up; and research capacity development	9 (15%)
Promotion/external Relations	Public relations; creating a development office; networking	9 (15%)

multiple goals possible in one Fellowship

Nine Fellows (15%) mentioned that the Partner goals were undefined or left entirely to the Fellow to develop, and 21 Fellows (35%) described how the goals were redefined or expanded during the Fellowship. For example, one Fellow remarked, "Once they realized I could do more they asked me to help with research. I wrote grant proposals and one was approved." Another stated "I sat down and said, 'I think I can do more' and wrote a whole set of goals ... and I added indicators for success."

On the other end of the spectrum were Fellowships that were overly ambitious, with too many tasks specified for the time and resources available or skill capacity of the Fellow. One Fellow was given a "laundry list that would have taken someone three years," while another mentioned partner goals changed "on a daily basis". Four Fellows mentioned that they narrowed the scope of the goals during the Fellowship.

### Fellows' perceptions of accomplishments

The diversity in Fellowship goals made it difficult to evaluate the Fellowships against a common standard. Instead, we focused on documenting Fellows' perceptions of goal accomplishment, perceived effects of the Fellowship expressed by Partners, and observed effects or documentation available during our site visits.

First, we asked Fellows to report on how well they thought they had achieved Partner Organization goals (see Table 2). Twenty-eight Fellows (46%) believed they had achieved "all" Partner goals, while an additional 20 (33%) reported having achieved "most" goals. Only one Fellow (2%) felt none of the Partner's goals for the Fellowship had been achieved. Where Fellowship goals were modified during the Fellowship, we asked Fellows to evaluate their accomplishment against the amended goals.

**Table 2:** Fellows reporting on goal accomplishment

Proportion of NGO goals achieved:	Number	%
All	28	46%
Most	20	33%
Some	8	13%
Few	2	3%
None	1	2%
Don't know	2	3%

Through open-ended questions, we sought to understand what Fellows believed were the most important effects that their Fellowships had on Partners. Fellows commonly mentioned three types of benefits:

▪ improved morale and increased pride in work on the part of Partner Organization staff;

▪ shifts to more strategic or business thinking (i.e. changing how Partners' viewed their target populations, project priorities, or systems needs);

▪ stronger technical or management capacity of the Partner Organization (i.e. enabling a new activity or improving existing activities).

As shown in the next section, data collected from NGO respondents supported these findings.

### Partner perceptions of accomplishments

Interviews with respondents at Partner Organizations provided examples of how Fellows helped organizations develop greater professional pride, confidence and self-efficacy, a "systems way of thinking" and business approach to management, and new technical skills. NGO perceived benefits from the Fellowships are discussed in three areas: skills transfer, operational improvements, and attitudes.

According to Partner Organization respondents, Pfizer Fellows transferred skills in the areas of medical practice, nursing, pharmacology, laboratory science, computer technology, facility and equipment maintenance, financial systems, epidemiology and biostatistics, marketing, program evaluation, and design and management of clinical research trials. These skills were transmitted in a variety of ways. Some Fellows were assigned to academic institutions, where they helped local faculty with curriculum development and actually taught classes and seminars on topics not previously covered. Others provided on the job training, often as part of an assignment to develop a particular product or operational improvement. Fellows sometimes taught computer skills by showing counterparts how to use an office application such as PowerPoint for their work, or by teaching a statistics application.

On the clinical research side, Fellows helped to write research grant proposals, design clinical trials, and train staff in proper trial procedures. For example, respondents in one Partner Organization attributed a newfound ability to compete for grants (demonstrated by two grants submitted and one funded) to the work of a Fellow. In another organization, a Fellow set up clinical trial management systems that organized and streamlined work, and could easily be modified to accommodate different study protocols in the future.

Turning to operational improvements in administrative and clinical care systems, most Partner Organization respondents readily cited operational changes that came from the work of the Fellows. Often the Fellow worked with an individual or team, using a combination of technical knowledge and management techniques to design and implement changes. For example, in a large, private non-profit teaching hospital, a sequence of two Fellows with extensive pharmacy operating experience worked with senior pharmacy staff to design a new system for recording drug procurement transactions; a drug pricing system with lower mark-ups on expensive drugs to make these more affordable to some seriously ill patients; and staffing changes based on standard productivity measures to reduce waiting time and increase client through-put. The Fellows also helped pharmacy staff to work with the Information Technology department to integrate necessary changes into the hospital's computerized systems. The Pharmacy Director described the effect of the new procedures: "I used to spend up to four hours a day signing documents. I brought them with me to meetings. When I signed one pile, it was replaced by another. Now it is reduced to nearly nothing. [The Fellow] has freed me to develop the Department." Examples of other operational changes are shown in Table 3.

**Table 3:** Examples of accomplishments of fellowships

**Organization**	**Operational Change**	**Effect**
NGO scientific research and clinical care facility	Creation of preventive maintenance schedules and routines, including budgets for necessary replacement parts	Equipment in working order, with sufficient budget for repairs and maintenance
NGO clinical research organization	Training in research methods, design of clinical trial management systems, grant writing assistance	Submission of research grants obtaining new sources of funding
NGO service delivery organization	Development of a financial system to track cost and budget at multiple new facilities, and to meet donor reporting requirements	Increased services delivered and revenues received
NGO teaching hospital	Revision of pharmacy operating procedures and pricing policies; creation of staff productivity measures; quality improvement teams	Reduced paperwork; better aligned prices with cost and patient ability to pay; reduced waiting times

A wide variety of work products were produced by the Fellows, including standard operating procedures, policy manuals, problem analysis documents (e.g. SWOT analysis and flow charts), and treatment protocols. In some cases, these depended on the Fellow's professional expertise. At one Partner, codification of procedures enabled the Organization to apply for formal accreditation for its services. Some Partner Organizations were able for the first time to address issues (such as development of a public relations strategy) because the Fellow provided the first available expertise in this area.

In other cases, the product was not dependent on a unique skill. The Fellow had the time and writing ability to prepare documents within the professional competence of existing staff, but which NGO personnel did not have the time to prepare. Fellows also taught counterparts how to write better reports. "I learned how to write a report systematically....this is a format we can use for all our work," said one counterpart.

In a few cases, a Fellow provided energetic volunteer assistance, relatively unrelated to the Fellow's expertise, in carrying out the mission of the Partner Organization. The Fellow's determination and hard work enabled the Organization to improve or expand its service while the Fellow was present. For example, one volunteer organized local artists to decorate drab pediatric wards with bright murals. Another organized a major community clean-up operation, even raising some of the funds for equipment. The community was still cleaner than usual six months after the Fellow's departure. However, with no clean up tools regularly available, it may be difficult to maintain the community spirit the Fellow engendered.

Finally, although the impact is more difficult to quantify, Partner Organization personnel offered examples of ways in which Fellows changed attitudes and stimulated new approaches to problem solving. For example,

#### Networking

Fellows demonstrated the value of networking and reaching out to development partners. To better serve an impoverished rural population, one Fellow suggested that the NGO approach the local government health department to urge expansion of in-village HIV counselling and testing. These negotiations led the government to take action and dedicate resources to in-village VCT, rather than forcing villagers to individually incur transport costs to reach the urban VCT center. In another country, a Fellow introduced NGO staff to a government office which had grant revenue and was looking for projects to fund, while also connecting the NGO to a local University looking for opportunities to involve student and faculty volunteers in community service projects.

#### Business perspective

Fellows convinced Organizations of the need to measure and continuously evaluate interventions, with Fellows actually working to draw up evaluation plans. Fellows suggested ways to measure the impact of social marketing programs, and to assess productivity and objectively assess staff performance. One respondent explained, " [The Fellow] analyzed work efficiency. We didn't know how to manage our own productivity before. We did not know how to justify new staff. Now we can justify.....Definitely, the changes will be lasting."

#### Individual initiative

Fellows stimulated initiative in their counterparts. One respondent said " [The Fellow] taught us to be responsible. We used to wait for higher authority to tell us what to do. [The Fellow] told us that the whole responsibility should be ours. Our talents, feelings, ideas can be brought out in our work.... Now, we don't postpone things so much, and it helps to avoid having things clump up. When we start something, we finish it."

#### Teamwork and time management

Fellows encouraged counterparts to work in teams, and to plan and monitor their own work. Work organization often improved as a result of the Fellows' example. An employee in one organization noted: "I learned time management. We would have a plan that said when we were to do things, and then we did them according to the plan. That wasn't the way we worked before."

### Fellowship problems

A few Fellowships ran into problems because of personality clashes, lack of effort on the part of the Fellow, and culture shock. These problems may be mitigated through careful selection, training, and matching of Fellows to Organizations. Other problems generally contributing to the lack of success in achieving Fellowship goals included:

▪ The Fellow did not have the skill set expected by the host organization;

▪ The Terms of Reference for the Fellowship were vague or inaccurate;

▪ Critical prerequisites–computer software or hardware, or a key counterpart–were not available when the Fellow arrived;

▪ Access to critical counterparts in the organization was too limited. The chosen counterpart may have been too high or too low in the organization;

▪ Counterparts were not able to prioritize the Fellow's assignment. This may have been a result of excessive routine work load, poor motivation, or the failure to involve the work unit in defining the need for a Fellow;

▪ The Partner Organization did not have the management or financial flexibility to implement the Fellow's recommendations.

### Typology of fellowship impact

A key question of concern to Pfizer and USAID was whether the Global Health Fellows Program was having an impact on service expansion in the countries and organizations where it was working. To answer this question, the research team attempted to define a typology delineating three main levels of Fellowship impact. The typology is presented in Table 4.

**Table 4:** Typology of Pfizer corporate volunteering Fellowships

**Type 1 **Accomplishments limited. Fellow provided volunteer assistance not requiring fellow's professional training and not building on expertise acquired through employment at Pfizer. The skills applied are available in-country or from many other international volunteers. Clients of the partner organization may have benefited from Fellow's personal efforts, but there was little or no permanent change in the ability of the partner organization of its staff to deliver services.

**Type 2 **Fellow provided technical expertise or training, based on fellow's professional training or Pfizer acquired expertise, which resulted in upgrading the skills of staff in the partner organization, or design of tools/methods for future changes. This may include creation of teaching curriculum, clinical guidelines and protocols, standard operating procedures as well as on-the-job and classroom training. No obvious effect on volume of services, but provided ground work for future improvements or increases in services, and quality may be improved.

**Type 3 **Using professional skills and/or Pfizer acquired expertise, Fellow worked with counterparts to introduce an operational or managerial improvement that will result in expanding services of the organization. Used skills or expertise not generally available in country. The quantity of service is increased directly, or as a result of improved efficiency/lowered unit cost of a service. May include changes in the organization and work ethic of a partner organization if these are directly attributable to the Fellow's effort and translate into sustainable service expansion.

By reviewing data from Fellows, Supervisors, and (in the five countries visited) Partner Organizations, the research team was able to place most Fellowship assignments within this typology. As the typology model was created at the end of the study, we did not measure the constructs explicitly during the research process; consequently, the model's reliability is not yet established. Despite this limitation, we present the typology to illustrate a potential avenue for empirical evaluation of corporate volunteering programs in the future.

A change in operations based on the Fellow's work, and which facilitated the sustained expansion of services, was classified as Type 3. Thirty-one percent of Pfizer Fellowships fell into this category. For example, one Fellow's work resulted in an increase in the volume of CD4 tests [a test of the immune function, used for HIV/AIDS care] performed by the Partner Organization, from 100 to 300 a day, while simultaneously increasing test quality and reliability. At another Partner Organization, a Fellow created an accounting software application for revenue reporting which saved staff six working hours per week while accommodating a tripling in the amount of revenues recorded.

Fellowships were classified as Type 2 if they involved transfer of skills and expertise through teaching, or the development of documents such as clinical protocols. These capacity building activities were perceived as important building blocks toward service expansion, although for these Fellowships there was less evidence that expansion had occurred yet. Almost half of Fellowships (48%) were categorized as Type 2.

A volunteer effort not based on the special skill and expertise of the Pfizer employee, and not transferring scarce skills to the Partner, was classified as Type 1.

Thirteen percent of Fellowships fell into Type 1, while an additional 8% of Fellowships lacked sufficient data for classification.

The research team found an increased number of Type 3 Fellowships in later rounds of the program, although the difference was not statistically significant. If real, such a difference could reflect the increased attention to defining specific goals and objectives for the Fellow's scope of work early in the assignment, which involved more communication with Partner Organization staff prior to the Fellow's arrival. When comparing current versus returned Fellows, we did not observe differences in Fellowship type, and there were no significant differences between Fellowship performance according to the region of the world where the Fellow worked. We did note an association between Fellowship length and performance, with longer Fellowships scoring higher. While the association did not reach statistical significance, this finding seems important. A possible confounder could be program timing: more of the shorter length Fellowships were held in the first year of the GHF program, when the program design and management systems were still being refined.

## Discussion

The evaluation results raise important operational, strategic, and methodological questions. Operational issues are addressed first, followed by a discussion of more general strategic questions concerning the design of international corporate volunteering programs. The last section discusses methodological concerns, and suggests steps needed to improve future design and implementation of programs.

### Operational issues

Corporations seeking to contribute resources to global health and development through public-private partnerships using corporate volunteering should consider lessons learned from the Pfizer experience. For example, the Pfizer program demonstrates the need to define Fellowship assignments with specific technical assistance goals and tasks, in order to make the best match between volunteer employees and the Partner Organizations. Prerequisites for assignments (including necessary software or staff) should be in place before Fellows are sent, and assigned counterparts must have sufficient time and decision making authority to support the Fellowship goals.

As other researchers have noted, philanthropic partnerships in the developing world need to be sure that volunteers are prepared for different cultural and social decision making processes, language differences, and unfamiliar bureaucracy [13]. Pfizer has developed effective orientation programs for Fellows which could serve as a model for other companies. For example, over time, Pfizer has adjusted the security and health briefings to incorporate examples and experiences of Fellows in the field. These programmatic adjustments and training concerns are not unlike those needed to maximize other types of donor-funded or development bank technical assistance initiatives.

The research team was surprised by the large percentage of women participants in the Pfizer program, a figure disproportionate to Pfizer's distribution of professionals eligible for participation. Pfizer staff could not give reasons for this discrepancy, but it may be because women are more likely to participate in any type of volunteering effort [22]. In addition, the program is not explicitly linked to management development programs at Pfizer, which may make it appear to be a "side track" from a standard corporate career advancement track.

### Strategic issues

Consideration of gender imbalance leads to more strategic considerations of program design. One of these issues is where corporate volunteering programs should be placed in the corporate structure, and their dual role as a corporate social responsibility initiative and a human resources development program. Discussions are currently underway at Pfizer to integrate the GHF program with other management development initiatives, in part to mitigate gender imbalance but also to increase the sustainability of the program by making it less reliant on the support of any particular corporate "champions". Pfizer is also considering adding some shorter-term volunteering opportunities in order to appeal to higher level managers and executives who cannot be away from their job responsibilities for longer periods. The higher-level volunteers would be placed in relatively complex local organizations which have previously hosted Fellows for longer periods and shown capacity to integrate technical assistance effectively. This approach would maximize the probability of high impact assignments within the shorter time frame.

Another strategic issue raised by the evaluation results is program design. The study raises the question of whether Pfizer's strategy of tailored technical assistance working with myriad local Partner Organizations is the best corporate investment in building human resource capacity in developing countries. A key factor in operation of Pfizer's corporate volunteering program has been the lack of a "cookie cutter" approach to technical assistance. Pfizer chose not to "project-ize" the Fellowship assistance by defining strategic results areas in advance. Instead, the program identifies local partners and starts from where they are, addressing the unique needs of each individual organization. This strategy creates management challenges. As the Partner Organizations vary in size, mission, ownership, and years of experience, this means each Fellowship may set unique goals, apply different approaches, and engage in different activities with varying outputs. While Pfizer has made efforts to encourage internal sharing of technical approaches and dissemination of lessons learned, there is still a risk of Fellows "reinventing the wheel" in developing new systems when these exist in similar programs with which the volunteer was not familiar.

An alternative strategy to improve program effectiveness is to focus on a more homogeneous set of partnerships or technical assistance goals. Research sponsored through the Brookings Initiative's Initiative on International Volunteering may provide some guidance in this regard. The Brookings Initiative's Corporate Engagement Working Group has commissioned a white paper on international corporate volunteering in an effort to document best practices for selection and deployment of volunteers and evaluation of program impact. Initial findings suggest that some companies are focusing capacity-building assistance on specific types of organizations or interventions. For example, the multinational medical technology company BD (Becton Dickinson Corporation) worked in collaboration with the Zambian Catholic Medical Mission Board to field 10 corporate volunteers for a two-week assignment in 2005 and 2006 [23,24]. The volunteers installed laboratory equipment and trained staff at five local facilities. While the sustainability of this program or others like it is not well documented, more focused programs such as BD's have intuitive appeal. By reducing the breadth of capacity building assistance, the program may be able to offer more relevant and tailored technical assistance, and can train and orient volunteers more efficiently. A "hybrid" design approach (narrowly targeted but also open-ended) is illustrated by the public-private partnership between the international transport corporation TNT and the UN World Food Program. This program is focused on the function of delivering World Food Program supplies and aid during disasters, but the capacity building technical assistance provided by TNT is varied and tailored to the specific field office being assisted and its specific mission, goals, history and resources [20]. Pfizer is currently considering changes to their international volunteering program to sharpen the strategic focus.

More controversial is the challenge of deciding which organizations to support through corporate volunteering. Sending a succession of Fellows to well-managed, relatively complex organizations that have made effective use of previous Fellowships may increase impact; yet smaller organizations also need capacity building help, especially since these organizations can play an integral role in achieving goals of equity and access for marginal populations. One possible strategy to include smaller organizations might be to encourage the creation of networks of Partner Organizations within a given area or country, allowing large and small organizations to share management tools and encouraging collaborative improvement efforts.

### Methodological issues

A final question concerns the types of evaluation tools and methods which are needed to measure the sustained impact on the organizations receiving assistance. The GHF program experience suggests a need to sensitize employee volunteers to the benefits of measuring program impact and documenting results. A follow-on research study funded by the United States Agency for International Development (USAID) Global Health Office and Pfizer is designed to collect measures of capacity building impact, including more extensive development of an impact typology such as the one presented here. The study will also measure factors which may influence program impact, including Fellow characteristics, Partner Organization level of development, and features of the Fellowship itself (e.g. length, tasks, etc.).

Future evaluations might also try to systematically survey all groups (Fellows, Supervisors, and Partners) and pose questions in such a way that they cross-refer. This would allow comparison of perceptions of impact. Discrepancies might help identify specific ways to improve the matching between Fellows and Partners, and better coordinate the collaborations and mutual expectations.

### Benefits for corporate partners

What did Pfizer get out of the program, and what benefits might other corporations obtain by adopting public-private partnerships based on corporate international volunteering? In recent years, public expectations have grown regarding the role business should be playing in global health. People expect private companies to be involved in increasing access to services as a duty of corporate citizenship. Observers have noted that companies can use philanthropy as a way to rebuild eroding trust and establish the public acceptance needed to stabilize their marketplace [22,25]. Interviews with key opinion leaders in several countries suggest that Pfizer's program may have had a positive influence on the company's reputation, although quite a few opinion leaders were unaware of the program, or did not know that the Fellows were associated with Pfizer.

The evaluation found that the Program had a positive impact on the Fellows themselves, their professional development, and the pride and satisfaction of the Fellows' work groups at Pfizer. Fellows reported believing that their experience dealing with sometimes inadequate resources, uncertainty, and cultural differences will make them better, more flexible managers. They expect the Fellowship will give an advantage in future assignments and promotions. Seventy-seven percent of Fellows felt the effect on their professional development was positive. Pfizer's commitment to the GHF Program made the Fellows (and their co-workers) proud to be a Pfizer employee. These findings are described in more detail elsewhere [26].

This evaluation has several limitations. First, while we made several attempts to contact Pfizer Fellows who had subsequently left the company, we were only able to contact one out of the nine Fellows no longer employed by Pfizer. It is possible that these Fellows had different experiences from those who are still at Pfizer, though it is unlikely that this has affected the information reported by Partner Organizations. Secondly, the evaluation was only able to conduct site visits in five countries, including 60% of Fellowship experiences. The experiences of Partner Organizations not visited may be different. In addition, although efforts were made to assure informants that responses were confidential and anonymous, it is possible that some respondents were reluctant to share criticism. Finally, our typology of Fellowship impacts was created retrospectively and must be interpreted with caution. Further research is needed to test the reliability of this typology.

Pfizer provided partial funding for this study, as described in the Competing Interests section. Pfizer staff did not have editorial control or review the manuscript before submission, mitigating the potential for bias in reporting results.

## Conclusion

Public-private partnerships and corporate philanthropy have significant resources to leverage toward expanding health services in developing countries. In recent years, corporate philanthropy has shifted to "interdependent philanthropy", with the dual goal of addressing social problems while also furthering the company's strategic interests and expanding core business [13]. Strengthening health services in poor countries is an area where interdependent philanthropy can make a difference. More research is needed to develop frameworks and methods for evaluation of international corporate volunteering programs. The lessons learned in evaluating such programs can help direct future investments to build capacity in sustainable ways.

## Competing interests

Pfizer Corporation provided 50% of the funding for the study. The research was also supported by federal funding through the United States Agency for International Development, as described in the Acknowledgements. At the time of the study, FF, TV, SR, and KM were full-time employees of Boston University, while PC was a consultant to Boston University. Pfizer staff did not have any editorial control on the manuscript and did not see a copy prior to submission. Pfizer funding did not pay for the preparation of the manuscript.

## Authors' contributions

TV participated in study design, administered surveys, conducted site visits in India, analyzed site visit data, and led the writing of the manuscript. SR participated in study design, administered surveys, conducted site visits in Ghana, performed qualitative data analysis, and helped to write the manuscript. KM administered surveys, coordinated the study, and performed statistical analysis. PC participated in study design, administered surveys, and conducted site visits in South Africa and Kenya. FF conceptualized the study, participated in its design, administered surveys, conducted site visits in Kenya and Uganda, analyzed site visit data, and helped to write the manuscript. All authors read and approved the final manuscript.
